# Intraventricular Hemorrhage Burden and Treatment-Specific Symptomatic Vasospasm After Aneurysmal Subarachnoid Hemorrhage

**DOI:** 10.3390/diagnostics16152330

**Published:** 2026-07-25

**Authors:** Shin-Woong Ko, Byeong Jin Ha, Sang Mook Kang, Jin Hwan Cheong, Je Il Ryu, Yu Deok Won, Myung-Hoon Han

**Affiliations:** Department of Neurosurgery, Hanyang University Guri Hospital, Guri 11923, Republic of Korea; pakky100@naver.com (S.-W.K.); bbddock@hanmail.net (B.J.H.); ksmuk0410@naver.com (S.M.K.); cjh2324@hanyang.ac.kr (J.H.C.); ryujeil@hanyang.ac.kr (J.I.R.)

**Keywords:** aneurysmal subarachnoid hemorrhage, intraventricular hemorrhage, Graeb score, symptomatic vasospasm, surgical clipping, endovascular coiling, cerebrospinal fluid diversion

## Abstract

**Background/Objective:** The modified Fisher scale classifies intraventricular hemorrhage (IVH) as present or absent, potentially obscuring meaningful vasospasm risk differences. This study aimed to identify the IVH burden threshold, measured by the Graeb scale, at which symptomatic vasospasm risk increases after aneurysmal subarachnoid hemorrhage (SAH) and whether this association differs by treatment modality. **Methods:** This retrospective single-center study included aneurysmal SAH patients undergoing microsurgical clipping or endovascular coiling between 2015 and 2025. IVH was graded using the original Graeb scale (range, 0–12) and categorized as no IVH (0), grades 1–3, 4–7, or ≥8. The outcome was symptomatic vasospasm. Multivariate logistic regression and exploratory subgroup analyses were performed. **Results:** Among the 307 patients analyzed (clipping, *n* = 224; coiling, *n* = 83), symptomatic vasospasm occurred in 63 (20.5%). On multivariate analysis, an IVH Graeb score ≥ 4 was the only independent predictor of symptomatic vasospasm (odds ratio, 4.32; 95% confidence interval, 2.10–8.87; *p* < 0.001). Exploratory subgroup analysis showed the clipping-coiling difference was most pronounced in Graeb 4–7 (18.2% vs. 62.5%; *p* = 0.008), with no significant differences in remaining categories. However, the treatment × Graeb-category interaction was non-significant (*p* = 0.066). **Conclusions:** An IVH Graeb score ≥ 4 independently predicted symptomatic vasospasm after aneurysmal SAH. The treatment-specific divergence observed in the Graeb 4–7 subgroup was exploratory and hypothesis-generating, suggesting that early cerebrospinal fluid diversion may warrant particular consideration when endovascular coiling is performed in patients with moderate IVH.

## 1. Introduction

Cerebral vasospasm remains one of the most significant causes of morbidity and mortality following aneurysmal subarachnoid hemorrhage (SAH) [1]. The modified Fisher grading scale, which incorporates the presence of intraventricular hemorrhage (IVH) as a binary variable, is the most widely used tool for predicting vasospasm risk [2]. However, this grading scale categorizes IVH simply as present or absent and does not account for the volume or distribution of intraventricular blood. Therefore, patients with markedly different IVH burdens may receive the same modified Fisher grade, potentially obscuring clinically meaningful differences in vasospasm risk.

Accumulating evidence indicates that IVH independently contributes to vasospasm through hemoglobin degradation products that exert spasmogenic effects on cerebral arteries [3]. Meanwhile, the Graeb scale provides a semiquantitative assessment of IVH severity across all four ventricles, offering a more granular measure than the binary classification of the modified Fisher scale [4]. Beyond the modified Fisher grade, several dedicated grading systems have been developed to quantify intraventricular and cisternal blood more precisely, including the LeRoux score [5], Hijdra scale [6], semiautomated IVH score [7], and modified Graeb score [8]; however, these systems were validated predominantly against outcomes such as acute hydrocephalus, functional recovery, or mortality rather than vasospasm. However, the specific IVH burden threshold at which the risk of symptomatic vasospasm significantly increases has not yet been defined. Angiographic vasospasm (radiographic arterial narrowing) must be distinguished from symptomatic vasospasm, which requires additional new neurological deficits. Symptomatic vasospasm overlaps with but is narrower than delayed cerebral ischemia (DCI), which also includes infarction not attributable to large-vessel spasm [9]. We use symptomatic vasospasm because our focus was the direct association between confirmed arterial narrowing and clinical deterioration.

The treatment modality may also modulate vasospasm risk independent of aneurysm obliteration. Microsurgical clipping inherently involves opening the Sylvian fissure and basal cisterns, fenestration of the lamina terminalis, and cisternal irrigation, all of which facilitate the early clearance of spasmogenic blood products [10]. In contrast, endovascular coiling does not provide intraoperative access to the subarachnoid space. Although this concept is well established, no prior study has identified the specific range of the IVH burden within which these treatment-specific differences in vasospasm risk manifest.

Therefore, this study aimed to determine the IVH burden threshold, as measured by the Graeb scale, at which the risk of symptomatic vasospasm increases significantly after aneurysmal SAH and to examine whether this association varies by treatment modality.

## 2. Materials and Methods

### 2.1. Study Design and Patients

**Figure 1:**
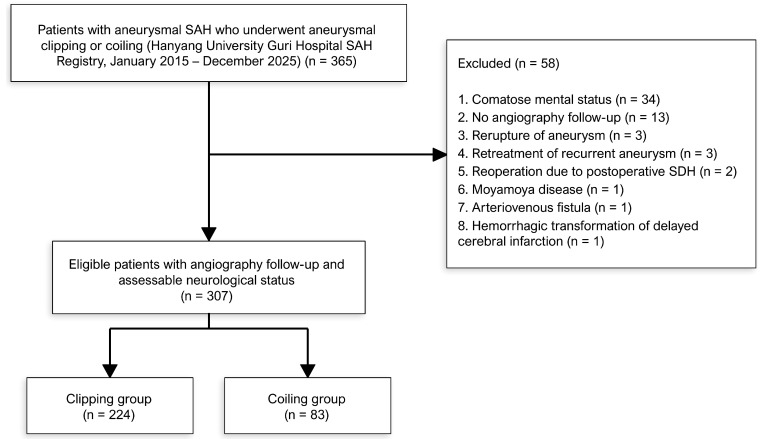
Patient selection flowchart. Of the 365 patients with aneurysmal SAH treated at Hanyang University Guri Hospital (January 2015–December 2025), 58 were excluded, leaving 307 patients (clipping, *n* = 224; coiling, *n* = 83) for analysis. Abbreviations: SAH, subarachnoid hemorrhage.

This study was a retrospective analysis of prospectively maintained data from the SAH Registry of Hanyang University Guri Hospital. We identified all consecutive patients (aged > 18 years) with aneurysmal SAH who underwent microsurgical clipping or endovascular coiling at the Hanyang University Guri Hospital between January 2015 and December 2025. Of the 365 patients initially identified, 58 were excluded for the following reasons: comatose mental status precluding neurological assessment (*n* = 34), absence of angiographic follow-up (*n* = 13), aneurysm re-rupture (*n* = 3), retreatment of a recurrent aneurysm (*n* = 3), reoperation due to postoperative subdural hematoma (*n* = 2), moyamoya disease (*n* = 1), arteriovenous fistula (*n* = 1), and hemorrhagic transformation of delayed cerebral infarction (*n* = 1). The remaining 307 patients (clipping, *n* = 224; coiling, *n* = 83) comprised the final cohort and were analyzed (Figure 1).

This study was approved by the Institutional Review Board of Hanyang University Guri Hospital and conducted in accordance with the Declaration of Helsinki. The requirement for informed consent was waived due to the retrospective nature of the study. All individual records were de-identified before analysis.

### 2.2. Surgical Treatment and Management of Aneurysmal SAH

Three faculty neurosurgeons (J.M.K., J.H.C., and Y.D.W.) performed all surgical and endovascular procedures throughout the study period [11,12], while two additional neurosurgeons (S.M.K. and S.-W.K.) joined the institution in 2024 and 2025. Microsurgical clipping was the predominant treatment modality used throughout the study period. Although endovascular coiling was performed on a limited basis before 2020, the utilization of this technique increased substantially following the arrival of Y.D.W. in 2020, who thereafter performed the vast majority of the coiling procedures. All neurosurgeons who performed microsurgical clipping were trained by a single senior surgeon (J.M.K.) at the same institution, and all operators who performed endovascular coiling were trained by a single interventionalist (Y.D.W.). This unified mentorship lineage ensured a high degree of technical consistency across surgical and endovascular approaches among the operators, contributing to procedural homogeneity within this single-center cohort.

All patients were managed according to a standardized SAH treatment protocol that included intracranial pressure monitoring and control, nimodipine administration, and maintenance of euvolemia. The decision between microsurgical clipping and endovascular coiling was made by the treating neurosurgeon based on the aneurysm morphology, location, patient clinical status, and anatomical considerations. External ventricular drainage (EVD) or lumbar drainage was performed at the discretion of the treating neurosurgeon for acute hydrocephalus or cerebrospinal fluid (CSF) diversion based on a comprehensive assessment, including IVH burden, ventricular dilatation, obstructive hydrocephalus, and the clinical status of the patient. Ventriculoperitoneal shunt placement was considered in patients who developed shunt-dependent hydrocephalus during the follow-up.

### 2.3. Definition of Vasospasm

Angiographic vasospasm was defined as arterial narrowing confirmed on follow-up digital subtraction angiography, CT angiography, or transcranial Doppler ultrasonography, irrespective of clinical symptoms [13]. Symptomatic vasospasm, the primary outcome of this study, was defined as the occurrence of a new focal neurological deficit (e.g., hemiparesis, aphasia, hemineglect, deterioration of motor responses, or new pupillary abnormality) or a decline in the level of consciousness, not attributable to other causes (e.g., rebleeding, hydrocephalus, metabolic derangement, infection, or seizure) in the presence of angiographic vasospasm [13]. This study used the term symptomatic vasospasm rather than DCI [14] because our primary focus was the direct association between angiographically confirmed arterial narrowing and clinical deterioration. Accordingly, the term symptomatic vasospasm was used consistently throughout this report to denote the primary outcome, whereas DCI was used only when referring to previous studies that specifically adopted DCI as their endpoint. Structured neurological assessments were performed at least twice daily by the neurosurgical team throughout the vasospasm risk period; when new deterioration was identified, prompt workup (including repeat CT, laboratory evaluation, and angiography) was performed to exclude alternative etiologies before attributing the deterioration to vasospasm. Follow-up vascular imaging was performed routinely in all patients during the vasospasm risk period (typically 3–14 days after SAH onset) as part of standardized surveillance, independent of clinical status, with additional imaging obtained whenever clinical deterioration occurred. Because patients without angiographic follow-up during this period were excluded a priori (*n* = 13; Figure 1), vascular imaging ascertainment was complete within the analyzed cohort, and this surveillance protocol was applied identically to the clipping and coiling groups.

### 2.4. Assessment of IVH

IVH severity was graded on admission computed tomography (CT) using the original Graeb scale [4]. All admission CT scans were independently reviewed by two faculty neurosurgeons (S.-W.K. and M.-H.H.) who were blinded to the clinical outcomes; discrepancies were resolved by consensus. The Graeb scale assigns scores of 0–4 for each lateral ventricle (0 = no blood, 1 = trace, 2 = <50% filled, 3 = >50% filled, 4 = filled and expanded by clot) and scores of 0–2 for the third and fourth ventricles (0 = no blood, 1 = blood present but ventricle not expanded, 2 = filled and expanded by clot), yielding a total score between 0 and 12 (Table 1 and Figure S1). For the categorical analysis, the Graeb score was classified into four tiers: no IVH (Graeb 0), grades 1–3, 4–7, and ≥8—the rationale for this categorization is presented in the Results section. This study used the original Graeb scale (range, 0–12) rather than the modified Graeb scale (range, 0–32) because, given our sample size, the finer granularity of the modified scale would result in excessive fragmentation of subgroups, potentially limiting the statistical power of categorical analyses. To confirm that this choice did not compromise discriminative accuracy, the two scales were compared using DeLong tests for paired receiver operating characteristic (ROC) curves (Figure S2).

### 2.5. Clinical and Radiographic Variables

Baseline demographic and clinical data were extracted from the medical records and operative reports, including age, sex, body mass index, hypertension, diabetes mellitus, smoking and alcohol history, Hunt and Hess grade at admission, and treatment modality (surgical clipping vs. endovascular coiling). Radiographic variables were assessed on admission using computed tomography, including the modified Fisher grade, aneurysm location, and presence of intracerebral hemorrhage (ICH). Management and procedural variables, including external ventricular drain placement, lumbar drain insertion, decompressive craniectomy, hydrocephalus development, and ventriculoperitoneal shunt placement, were also recorded (Table 2).

### 2.6. Statistical Analysis

Continuous variables are expressed as the mean ± standard deviation or median (interquartile range (IQR)) as appropriate, and categorical variables are presented as frequency (percentage). Differences between groups were compared using Student’s t-test or Mann–Whitney U test for continuous variables and the chi-square test or Fisher’s exact test for categorical variables.

The discriminative ability of the Graeb score to predict symptomatic vasospasm was evaluated using receiver operating characteristic (ROC) curve analysis, with the area under the curve (AUC) as a metric. Multivariate logistic regression analysis was performed to identify independent predictors of symptomatic vasospasm. The model included an IVH Graeb score ≥ 4 as the primary predictor, adjusted for age, sex, treatment modality, modified Fisher grade, Hunt and Hess grade, CSF diversion (EVD or lumbar drain), ICH, aneurysm location, hypertension, diabetes mellitus, smoking, and alcohol use. Exploratory subgroup analyses using logistic regression were conducted to examine whether the association between treatment modality and symptomatic vasospasm differed across the IVH Graeb score categories, and effect modification was assessed using the likelihood ratio test comparing the main effects and interaction models. For sensitivity analysis, the discriminative performance of the original and modified Graeb scales was compared using DeLong tests for correlated ROC curves. Additionally, sensitivity analyses were performed to test the robustness of the primary findings, including refitting the multivariable model with CSF diversion excluded to address potential collider bias, and evaluating the temporal shift in coiling utilization through era-stratified analyses and era- and year-adjusted logistic regression. Post hoc power for the Graeb 4–7 pairwise comparison and the treatment × Graeb-category interaction was computed using Cohen’s h and two-proportion z-approximation (R package pwr).

A value of *p* < 0.05 was considered statistically significant. All statistical analyses were performed using R software (version 4.3.3; R Foundation for Statistical Computing, Vienna, Austria).

## 3. Results

### 3.1. Baseline Characteristics

**Figure 2:**
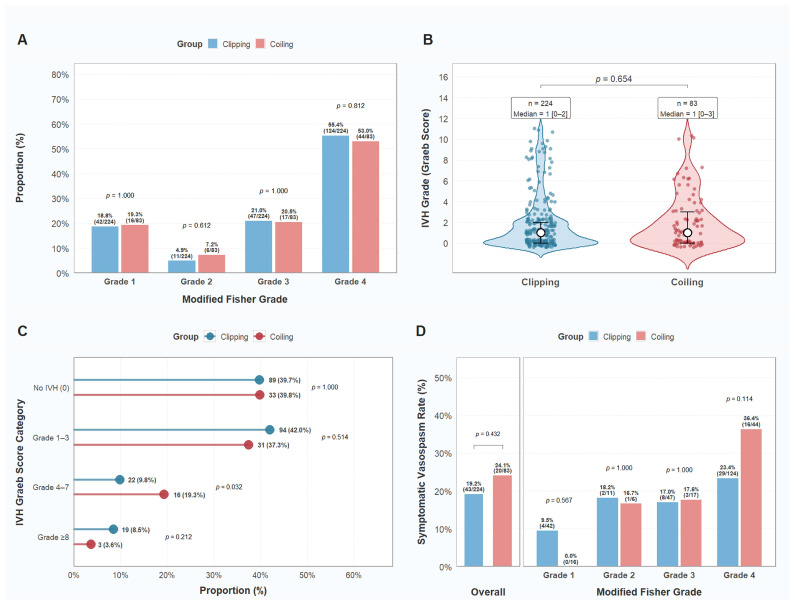
Comparison of hemorrhage burden and symptomatic vasospasm between the clipping and coiling groups. (**A**) Distribution of modified Fisher grades by treatment group. (**B**) Violin plots of the IVH Graeb score comparisons between treatment groups; circles and error bars represent medians and interquartile ranges. (**C**) Distribution of IVH Graeb score categories by treatment group. (**D**) Symptomatic vasospasm rates between treatment groups, shown for the total cohort and stratified by modified Fisher grade. The *p*-values were calculated using the chi-square test ((**A**,**D**) total cohort), Mann–Whitney *U* test (**B**), or Fisher’s exact test (**C**,**D**). Abbreviations: IVH, intraventricular hemorrhage.

A total of 307 patients with aneurysmal SAH were included in the analysis: 224 (73.0%) in the clipping group and 83 (27.0%) in the coiling group (Figure 1). The mean age was 57.7 ± 12.7 years, and 189 patients (61.6%) were female (Table 2). IVH was present in 185 patients (60.3%) with a median Graeb score of 1.0 (IQR, 0.0–3.0). The most common presentation was modified Fisher grade 4 (54.7%). The proportion of patients treated with clipping versus coiling was similar across all modified Fisher grades (Figure 2A), and the overall distribution of IVH Graeb scores did not differ significantly between the two groups (Figure 2B). However, the coiling group had a significantly higher proportion of patients in the Graeb grades 4–7 category than the clipping group (Figure 2C). Baseline demographics, comorbidities, and aneurysm characteristics are summarized in Table 2.

### 3.2. Symptomatic Vasospasm According to Treatment Modality

Symptomatic vasospasm occurred in 63 of the 307 patients (20.5%), with no significant difference between the clipping (43/224, 19.2%) and coiling (20/83, 24.1%) groups (*p* = 0.432; Figure 2D). When stratified by the modified Fisher grade, no significant between-group differences were observed for any individual grade. However, there was a non-significant trend toward a higher rate in the coiling group among patients with a modified Fisher grade of 4 (Figure 2D).

### 3.3. Association Between IVH Graeb Score and Symptomatic Vasospasm

**Figure 3:**
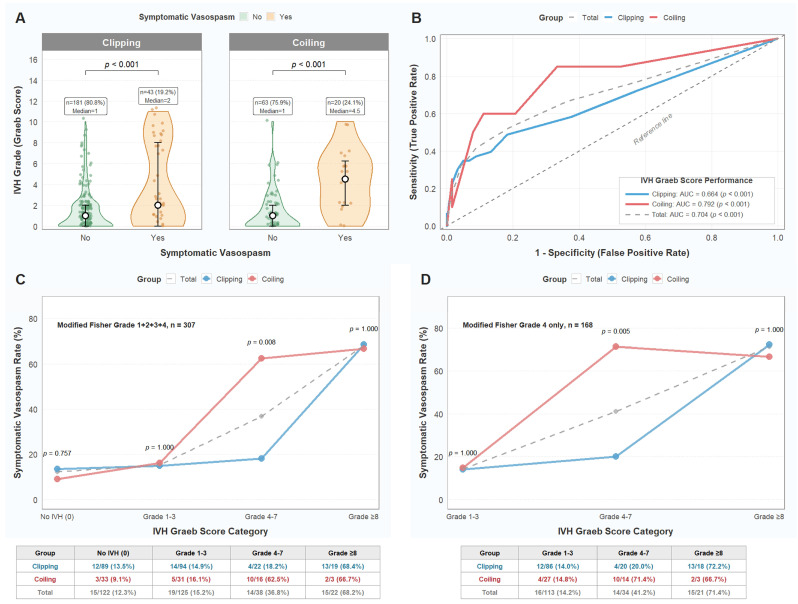
Association between IVH Graeb score and symptomatic vasospasm in the clipping and coiling groups. (**A**) IVH Graeb score distributions stratified by symptomatic vasospasm status within each treatment group; circles and error bars represent medians and interquartile ranges. (**B**) Receiver operating characteristic curves for the predicted symptomatic vasospasm using the IVH Graeb score, shown separately for the clipping, coiling, and total cohorts. (**C**) Comparison of symptomatic vasospasm rates between the clipping and coiling groups by IVH Graeb score category across all modified Fisher grades (*n* = 307). (**D**) Comparison of symptomatic vasospasm rates between the clipping and coiling groups by IVH Graeb score category in patients with modified Fisher grade 4 only (*n* = 168). The *p*-values were calculated using the Mann–Whitney *U* test (**A**) or Fisher’s exact test (**C**,**D**). Abbreviations: AUC, area under the curve; IVH, intraventricular hemorrhage.

IVH with varying Graeb scores was exclusively distributed in modified Fisher grades 2 and 4, with higher Graeb scores predominantly concentrated in grade 4 (Figure S3). Patients with symptomatic vasospasm had significantly higher Graeb scores than those without vasospasm in both the clipping (median, 2.0 vs. 1.0) and coiling (median, 4.5 vs. 1.0) groups (Figure 3A).

### 3.4. Treatment-Specific Symptomatic Vasospasm Risk by IVH Graeb Score Category

ROC analysis demonstrated that the Graeb score had a good discriminative ability for predicting symptomatic vasospasm, particularly in the coiling group (AUC, 0.792) compared to the clipping group (AUC, 0.664) (Figure 3B). A supplemental comparison of the original and modified Graeb scales showed that the finer 0–32 granularity of the modified scale distributed patients across seven progressively smaller strata (*n* = 122, 66, 65, 24, 16, 10, and 4 for modified Graeb score (mGS) = 0, 1–3, 4–7, 8–11, 12–16, 17–22, and ≥23; Figure S2A), while paired DeLong tests confirmed essentially identical discriminative performance in every subgroup (Total, 0.704 vs. 0.710, ΔAUC = +0.006, *p* = 0.409; clipping, 0.664 vs. 0.677, ΔAUC = +0.013, *p* = 0.020; coiling, 0.792 vs. 0.790, ΔAUC = −0.002, *p* = 0.881; Figure S2B), supporting the use of the original scale in our categorical analyses. When analyzed by the Graeb score category, the symptomatic vasospasm rate increased progressively, with higher Graeb scores in the total cohort (Figure 3C). Notably, the difference in symptomatic vasospasm rates between the clipping and coiling groups was most pronounced in grades 4–7 (clipping: 18.2% vs. coiling: 62.5%; *p* = 0.008; Figure 3C). This finding remained consistent when the analysis was restricted to patients with a modified Fisher grade of 4 (clipping, 20.0% vs. coiling, 71.4%; *p* = 0.005; Figure 3D). In contrast, no significant between-group differences were observed in the no IVH (*p* = 0.757), grade 1–3 (*p* = 1.000), or grade ≥ 8 (*p* = 1.000) categories. The rationale for adopting the four-tier Graeb score categorization (no IVH, grades 1–3, 4–7, and ≥8) is illustrated in Figure S4. Compared with the alternative Graeb score categorization schemes presented in Figure S4, this classification most clearly delineated the Graeb score range in which the coiling group exhibited a significantly higher vasospasm rate than the clipping group (grade 4–7), while simultaneously achieving the closest agreement in vasospasm rates between the two groups in the remaining categories (grades 1–3 and ≥8, both *p* = 1.000; Figure 3C). In the coiling group with Graeb grades 4–7, lumbar drain and EVD placement were performed in 50.0% and 12.5% of the patients, respectively; however, the symptomatic vasospasm rate in this subgroup remained elevated (Figure S5).

### 3.5. Multivariate Analysis and Treatment-Specific Risk by IVH Graeb Score Category

**Figure 4:**
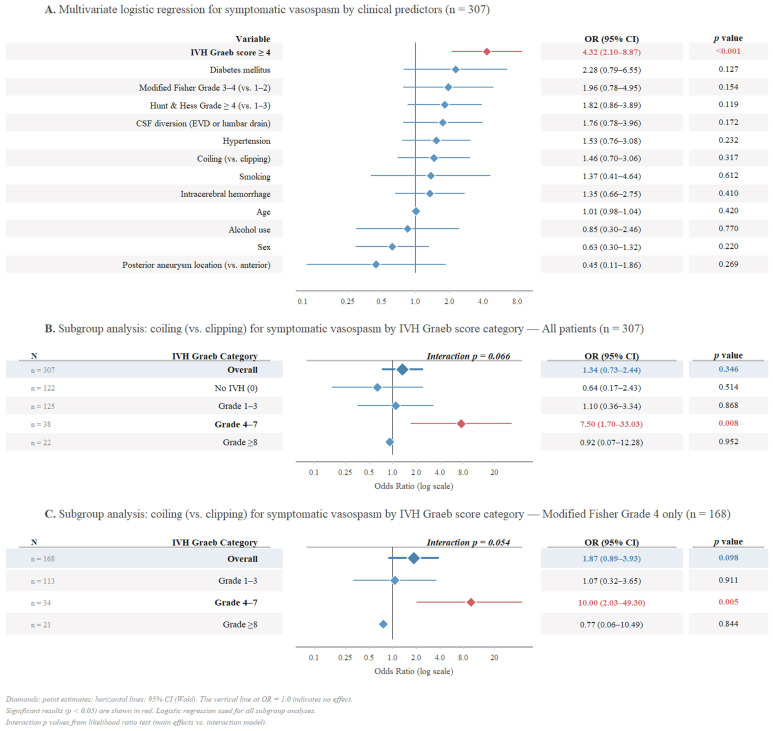
Multivariate logistic regression and subgroup analyses for symptomatic vasospasm. (**A**) Forest plot for the multivariate logistic regression analysis of the adjusted odds ratios for clinical predictors of symptomatic vasospasm in the total cohort (*n* = 307). (**B**) Subgroup analysis of the odds of symptomatic vasospasm for coiling versus clipping by IVH Graeb score category in all patients (*n* = 307). (**C**) Subgroup analysis restricted to patients with modified Fisher grade 4 (*n* = 168). Abbreviations: CI, confidence interval; CSF, cerebrospinal fluid; EVD, external ventricular drain; IVH, intraventricular hemorrhage; OR, odds ratio.

In the multivariate logistic regression analysis, an IVH Graeb score ≥ 4 was the only independent predictor of symptomatic vasospasm (odds ratio (OR), 4.32; 95% confidence interval (CI), 2.10–8.87; *p* < 0.001) after adjusting for treatment modality (coiling vs. clipping), modified Fisher grade (3–4 vs. 1–2), Hunt and Hess grade (≥4 vs. 1–3), CSF diversion (EVD or lumbar drain), ICH, aneurysm location (posterior vs. anterior), age, sex, hypertension, diabetes mellitus, smoking, and alcohol use (Figure 4A). As suggested by the divergent vasospasm rates between the treatment groups across the IVH Graeb score categories (Figure 3C), an exploratory subgroup analysis using logistic regression was performed to further examine this pattern. In the overall cohort (*n* = 307), the interaction between the treatment modality and Graeb category did not reach statistical significance (interaction *p* = 0.066; Figure 4B); however, the pattern observed in Figure 3C was reflected in the regression results. Within the Graeb grades 4–7 subgroup, coiling appeared to be associated with higher odds of symptomatic vasospasm than clipping (OR, 7.50; 95% CI, 1.70–33.03; *p* = 0.008), whereas no such difference was observed in the remaining categories. Moreover, a similar trend was observed when restricted to patients with a modified Fisher grade of 4 (*n* = 168), in which the divergence between the treatment groups was more pronounced (Figure 3D). The interaction term remained borderline nonsignificant (interaction *p* = 0.054; Figure 4C), but the point estimate in the Graeb grades 4–7 subgroup was larger (OR, 10.00; 95% CI, 2.03–49.30; *p* = 0.005), with no significant differences in the other subgroups. These findings were robust in the sensitivity analyses: effect estimates remained essentially unchanged after excluding CSF diversion from the multivariable model (Table S1), and treatment-specific divergence in the Graeb 4–7 subgroup persisted after adjustment for era or treatment year, with no evidence of era heterogeneity (Tables S2 and S3 and Figure S7). A post hoc power analysis further indicated that the pairwise Graeb 4–7 comparison was adequately powered (~80%), whereas the treatment × Graeb category interaction test was underpowered (~45–49%; Figure S8 and Table S4).

### 3.6. Proportion of Symptomatic Vasospasm Among Patients with Angiographic Vasospasm

Higher IVH Graeb scores were associated with increased rates of both angiographic and symptomatic vasospasms. Angiographic vasospasm rates rose from 49.2% in the no-IVH group to 81.8% in the grade ≥ 8 group, while symptomatic vasospasm rates increased from 12.3% to 68.2% over the same range (Figure S6A). The increase in symptomatic vasospasm was proportionally steeper, as reflected by the progressive rise in the symptomatic-to-angiographic vasospasm ratio: 25.0% in no IVH, 27.5% in grades 1–3, 58.3% in grades 4–7, and 83.3% in grades ≥ 8 (Figure S6B). When this ratio was compared between treatment groups, the clipping and coiling groups showed similar rates in the no IVH and grade 1–3 categories. In the grade 4–7 category, the coiling group showed a higher rate (71.4% (10/14) vs. 40.0% (4/10); *p* = 0.211), although the difference was not statistically significant (Figure S6C, D).

## 4. Discussion

This study examined the impact of IVH burden on symptomatic vasospasm after aneurysmal SAH and assessed whether this association varied according to the treatment modality. Two principal findings were observed.

### 4.1. IVH Graeb Score ≥ 4 as a Threshold for Symptomatic Vasospasm

The principal finding of this study was that an IVH Graeb score ≥ 4 was the only independent predictor of symptomatic vasospasm. This threshold phenomenon is underpinned by the dose-dependent toxicity of hemoglobin degradation products in the CSF. Indeed, oxyhemoglobin released from intraventricular clots induces vasoconstriction through nitric oxide scavenging, endothelin-1 release, free radical generation, and inflammasome-mediated interleukin-1 production [3,15]. Akeret et al. demonstrated a sigmoidal dose–response relationship between CSF hemoglobin and arterial vasoconstriction, with near-maximal contraction at approximately 10^−5^ M, above which endogenous scavengers (haptoglobin, hemopexin) are overwhelmed [16]. Graeb scores of 1–3, representing a low overall IVH burden, likely generate hemoglobin concentrations within the capacity of these endogenous defense mechanisms. In contrast, a Graeb score of ≥4, indicating a moderate-to-high IVH burden, such as significant filling of the lateral ventricles or extension into the third and fourth ventricles, likely exceeds this scavenging capacity, resulting in sustained exposure of cerebral arteries to unbound spasmogenic hemoglobin. Moreover, the intraventricular clot serves as a continuous reservoir that slowly releases heme products into the CSF circulation during the vasospasm risk period, as evidenced by reports of delayed vasospasm in patients with isolated IVH and no cisternal blood [17]. This reservoir effect amplifies the spasmogenic burden from the cisternal blood that is already present in patients with a modified Fisher grade 4. This reservoir effect also explains why IVH severity predicted symptomatic vasospasm independent of the modified Fisher grade in our multivariate model. In addition to large-vessel vasospasm, these hemoglobin degradation products increasingly penetrate the paravascular spaces at higher IVH burdens, impairing glymphatic clearance [18,19] and provoking microvascular spasm [20], neuroinflammation [15], and cortical spreading depolarization [3]. These processes are undetectable on conventional angiography and collectively erode the cerebrovascular reserve and deactivate autoregulatory compensation. Our data are consistent with this multilevel mechanism; the symptomatic-to-angiographic vasospasm conversion rate rose sharply from 27.5% at Graeb scores of 1–3 to 58.3% at Graeb scores of 4–7, despite only a modest increase in the incidence of angiographic vasospasm (55.2–63.2%). This dissociation suggests that large-vessel narrowing alone is insufficient to produce clinical deficits. Rather, the same degree of angiographic vasospasm becomes clinically devastating only when superimposed on a compromised microvascular environment created by a higher IVH burden.

### 4.2. Treatment-Specific Risk in the IVH Graeb 4–7 Subgroup

The most notable exploratory finding of this study was that the difference in symptomatic vasospasm between the clipping and coiling groups appeared to be confined to the Graeb scores 4–7 category (18.2% vs. 62.5%), with no difference observed in the lower (Graeb scores 0 and 1–3) or higher (Graeb scores ≥ 8) categories. Thus, we hypothesized that this treatment-specific divergence may reflect an “incidental CSF diversion” effect inherent to microsurgical clipping, which is absent in endovascular coiling. During microsurgical clipping, surgical access requires steps that collectively facilitate early CSF diversion: opening the Sylvian fissure and basal cisterns allows direct evacuation of cisternal clots; fenestration of the lamina terminalis provides drainage from the third ventricle; opening the Liliequist membrane restores communication between CSF compartments [10,21]. These maneuvers are performed for brain relaxation and surgical exposure, not for vasospasm prophylaxis; however, the cumulative effect of these maneuvers constitutes substantial early clearance of spasmogenic blood products from both the ventricular and subarachnoid compartments. Multiple studies have confirmed that intraoperative cisternal irrigation and clot removal during clipping reduce the incidence of vasospasm and DCI [10,22,23]. In contrast, endovascular coiling provides no intraoperative access to the subarachnoid or ventricular spaces; therefore, any CSF diversion in coiled patients must be deferred during the postoperative period. Although this mechanism was not directly measured in the present study, this distinction may be inconsequential when the IVH burden is low (Graeb scores 1–3), as natural CSF turnover can adequately clear limited blood products. At the opposite extreme (Graeb score ≥ 8), the overwhelming blood volume may exceed the clearance capacity of both natural circulation and surgical diversion, resulting in uniformly high vasospasm rates regardless of treatment modality. However, at the intermediate burden of Graeb scores 4–7, where natural clearance alone is insufficient but damage is not yet irreversible, early surgical CSF diversion via clipping possibly contributes to the lower symptomatic vasospasm rate observed in this subgroup; however, because intraoperative drainage volume and clot clearance were not measured, this explanation remains inferential. Observations from our data are consistent with this interpretation, although causation cannot be established. In the Graeb 4–7 coiling subgroup, the lumbar drain and EVD placement rates were 50.0% and 12.5%, respectively; however, symptomatic vasospasm remained at 62.5%. In the corresponding Graeb 4–7 clipping subgroup, the formal CSF diversion rates were substantially lower (lumbar drain, 9.1%; EVD, 4.5%); however, the vasospasm rate was only 18.2%. This observation may be consistent with the possibility that intraoperative CSF diversion during clipping could be more effective than postoperative external drainage alone, although this uncontrolled comparison involved small subgroups and must be regarded as hypothesis-generating. Meanwhile, direct surgical access to the basal cisterns during clipping provides the most immediate clearance of spasmogenic substances at the sites where they are most concentrated and at an intermediate IVH burden (Graeb scores of 4–7); this early clearance may help mitigate the progression to symptomatic vasospasm. Vanaclocha et al. further corroborated this concept by demonstrating that surgical clipping combined with cisternal urokinase administration yielded significantly lower vasospasm rates than endovascular treatment alone, particularly in high-grade SAH [22]. Nonetheless, our findings should not be interpreted as an argument against endovascular coiling in patients with an intermediate IVH burden, but rather as an emphasis on the importance of aggressive postprocedural CSF diversion when coiling is selected. Kwon et al. demonstrated that lumbar CSF drainage after endovascular coiling significantly reduced the incidence of clinical vasospasm, from 63.3% to 23.4% [24]. More recently, the EARLYDRAIN trial confirmed that early prophylactic lumbar drainage improved functional outcomes at 6 months and reduced the burden of secondary cerebral infarction after aneurysmal SAH, regardless of the treatment modality employed [25]. Taken together, these findings suggest that for coiled patients with an IVH with Graeb scores of 4–7, early and aggressive CSF diversion strategies should be strongly considered to bridge the gap left by the absence of intraoperative blood clearance.

Although the concept that microsurgical clipping may reduce vasospasm compared to endovascular coiling through intraoperative blood clearance is intuitively appealing, to our knowledge, no prior study has demonstrated the IVH range in which this protective effect actually manifests or the magnitude of the difference. Therefore, to our knowledge, the present study is the first to report that treatment-specific divergence may emerge within the Graeb 4–7 subgroup. However, as the interaction tests did not reach statistical significance, these findings should be regarded as hypothesis-generating and require validation in larger cohorts. Nonetheless, these results highlight the potential importance of considering IVH burden when anticipating symptomatic vasospasm risk across different treatment modalities.

This study had several limitations. First, its retrospective design was inherently susceptible to selection bias. Second, the overall sample size was modest, particularly in the Graeb 4–7 coiling subgroup, which limited the statistical power and generalizability of these findings. Relatedly, the single-center design and unified training lineage may limit the generalizability of our findings to institutions with different practice patterns. While this homogeneity improves internal consistency, it does not replace external validation, and our treatment-specific findings therefore require confirmation in multicenter cohorts. Third, the “incidental CSF diversion” effect of microsurgical clipping was inferred from the observed data rather than directly quantified, and the volume of intraoperative CSF drainage and clot removal was not systematically recorded; therefore, other surgery-related factors may have contributed to the lower vasospasm rate in the clipping group. Fourth, although endovascular coiling was performed on a limited basis before 2020, its utilization has subsequently increased substantially, potentially introducing a temporal bias. However, the standardized treatment protocol and unified training lineage at our institution help to mitigate this concern. Sensitivity analyses further showed that coiling patients’ baseline characteristics did not differ between the early (2015–2019) and late (2020–2025) eras and that the Graeb 4–7 divergence persisted after era- or year-adjustment, with a non-significant treatment × era interaction. Fifth, although verification bias was a potential concern, follow-up vascular imaging was performed routinely in all patients during the risk period, not only after clinical deterioration, and was applied identically in both groups, making differential outcome ascertainment an unlikely explanation. Sixth, the subgroup and interaction analyses were exploratory and limited by statistical power and multiplicity. In the post hoc analysis, the pairwise Graeb 4–7 comparison had approximately 80% power, whereas the treatment × Graeb interaction was underpowered (~45–49%) and required approximately 650–715 patients for confirmation. Because these analyses were also uncorrected for multiple comparisons, the associated *p*-values and the interaction itself should be regarded as hypothesis-generating rather than confirmatory. Because treatment allocation was non-randomized, residual confounding by indication cannot be fully excluded even after multivariable adjustment, which is a central reason why the treatment-specific finding is hypothesis-generating. Finally, residual confounding cannot be excluded, as several variables not captured in our registry could not be adjusted for, including antiplatelet or anticoagulant use, treatment timing after ictus, intraoperative and postoperative blood pressure management, and detailed aneurysm morphology beyond the location. These factors may affect both treatment selection and vasospasm risk, and should be considered when interpreting our treatment-specific findings. Future prospective multicenter studies with larger sample sizes and direct measurements of intraoperative blood clearance are warranted to validate these findings.

## 5. Conclusions

In this single-center retrospective study of 307 patients with aneurysmal SAH, a Graeb IVH score ≥ 4 was the only independent predictor of symptomatic vasospasm. The difference in vasospasm rates between clipping and coiling was confined to the Graeb 4–7 subgroup, suggesting that intraoperative CSF diversion inherent to microsurgical clipping may confer a protective advantage against intermediate IVH burdens. This treatment-specific observation should be regarded as exploratory and hypothesis-generating. These findings underscore the importance of IVH burden assessment in anticipating treatment-specific vasospasm risk and support the consideration of early aggressive CSF diversion strategies when endovascular coiling is performed in patients with moderate IVH.

## Figures and Tables

**Table 1:** Original Graeb scale for grading intraventricular hemorrhage severity on admission computed tomography.

Ventricle	Score 0	Score 1	Score 2	Score 3	Score 4	Maximum
Left lateral ventricle	No blood	Trace	<50% filled	>50% filled	Filled and expanded	4
Right lateral ventricle	No blood	Trace	<50% filled	>50% filled	Filled and expanded	4
Third ventricle	No blood	Blood present, not expanded	Filled and expanded	N/A	N/A	2
Fourth ventricle	No blood	Blood present, not expanded	Filled and expanded	N/A	N/A	2
Total Graeb score	Sum of all individual ventricle scores					0–12

Abbreviation: N/A, not applicable.

**Table 2:** Comparison of baseline characteristics between patients with and without symptomatic vasospasm.

Characteristic	Symptomatic Vasospasm (−)	Symptomatic Vasospasm (+)	Total	*p*
Number (%)	244 (79.5)	63 (20.5)	307 (100.0)	
Sex, female, *n* (%)	155 (63.5)	34 (54.0)	189 (61.6)	0.213
Age, mean ± SD, years	57.1 ± 12.8	60.0 ± 12.0	57.7 ± 12.7	0.102
BMI, mean ± SD, kg/m^2^	24.4 ± 3.5	24.5 ± 3.8	24.4 ± 3.5	0.833
Treatment, *n* (%)				0.432
Clipping	181 (74.2)	43 (68.3)	224 (73.0)	
Coiling	63 (25.8)	20 (31.7)	83 (27.0)	
Angiographic vasospasm, *n* (%)	108 (44.3)	63 (100.0)	171 (55.7)	<0.001
IVH, *n* (%)	137 (56.1)	48 (76.2)	185 (60.3)	0.006
Graeb score, median (IQR)	1.0 (0.0–2.0)	3.0 (1.0–7.0)	1.0 (0.0–3.0)	<0.001
Modified Graeb score, median (IQR)	2.0 (0.0–4.0)	6.0 (2.0–14.0)	2.0 (0.0–5.0)	<0.001
EVD, *n* (%)	18 (7.4)	17 (27.0)	35 (11.4)	<0.001
Lumbar drain, *n* (%)	18 (7.4)	13 (20.6)	31 (10.1)	0.004
Hunt and Hess grade, *n* (%)				<0.001
Grade 1	15 (6.1)	2 (3.2)	17 (5.5)	
Grade 2	140 (57.4)	15 (23.8)	155 (50.5)	
Grade 3	49 (20.1)	22 (34.9)	71 (23.1)	
Grade 4	37 (15.2)	24 (38.1)	61 (19.9)	
Grade 5	3 (1.2)	0 (0.0)	3 (1.0)	
Modified Fisher grade, *n* (%)				0.011
Grade 1	54 (22.1)	4 (6.3)	58 (18.9)	
Grade 2	14 (5.7)	3 (4.8)	17 (5.5)	
Grade 3	53 (21.7)	11 (17.5)	64 (20.8)	
Grade 4	123 (50.4)	45 (71.4)	168 (54.7)	
Aneurysm location, *n* (%)				0.839
ACA	92 (37.7)	26 (41.3)	118 (38.4)	
MCA	64 (26.2)	12 (19.0)	76 (24.8)	
ICA	15 (6.1)	4 (6.3)	19 (6.2)	
PCOM	58 (23.8)	17 (27.0)	75 (24.4)	
VBA	15 (6.1)	4 (6.3)	19 (6.2)	
ICH, *n* (%)	58 (23.8)	25 (39.7)	83 (27.0)	0.018
Decompressive craniectomy, *n* (%)	42 (17.2)	20 (31.7)	62 (20.2)	0.017
Hydrocephalus, *n* (%)	140 (57.4)	46 (73.0)	186 (60.6)	0.034
VP shunt, *n* (%)	24 (9.8)	15 (23.8)	39 (12.7)	0.006
Hypertension, *n* (%)	78 (32.0)	25 (39.7)	103 (33.6)	0.314
Diabetes mellitus, *n* (%)	15 (6.1)	11 (17.5)	26 (8.5)	0.009
Alcohol use, *n* (%)	37 (15.2)	10 (15.9)	47 (15.3)	1.000
Smoking, *n* (%)	26 (10.7)	9 (14.3)	35 (11.4)	0.558

Abbreviations: ACA, anterior cerebral artery; BMI, body mass index; EVD, external ventricular drain; ICA, internal carotid artery; ICH, intracerebral hemorrhage; IQR, interquartile range; IVH, intraventricular hemorrhage; MCA, middle cerebral artery; PCOM, posterior communicating artery; SD, standard deviation; VBA, vertebrobasilar artery; VP, ventriculoperitoneal.

## Data Availability

The datasets generated and analyzed during the current study are not publicly available due to privacy and ethical restrictions but are available from the corresponding author upon reasonable request.

## Supplementary Materials

The following supporting information can be downloaded at https://www.mdpi.com/article/10.3390/diagnostics16152330/s1. Figure S1: Original Graeb scoring system for intraventricular hemorrhage grading; Figure S2: Comparison of original and modified Graeb scales for the symptomatic vasospasm prediction; Figure S3: Distribution of IVH Graeb scores by modified Fisher grade; Figure S4: Sensitivity analysis of symptomatic vasospasm rates using alternative IVH Graeb score categorizations; Figure S5: Cerebrospinal fluid diversion procedures by IVH Graeb score category in the clipping and coiling groups; Figure S6: Angiographic and symptomatic vasospasm rates and the symptomatic-to-angiographic vasospasm ratio by IVH Graeb score category; Figure S7: Sensitivity analyses addressing CSF-diversion adjustment and the temporal shift in endovascular practice; Figure S8: Summary of the post hoc power analysis; Table S1: Multivariable logistic regression for symptomatic vasospasm — primary model vs. sensitivity model excluding CSF diversion (*n* = 307); Table S2: Baseline characteristics of coiling patients by era; Table S3: Sensitivity analyses for the temporal shift in treatment practice (*n* = 307); Table S4: Post hoc power analysis for the Graeb 4–7 pairwise comparison and the treatment × Graeb-category interaction (*n* = 307).

## Abbreviations

The following abbreviations are used in this manuscript:

AUC	area under the curve
CI	confidence interval
CSF	cerebrospinal fluid
CT	computed tomography
DCI	delayed cerebral ischemia
EVD	external ventricular drain
ICH	intracerebral hemorrhage
IQR	interquartile range
IVH	intraventricular hemorrhage
OR	odds ratio
ROC	receiver operating characteristic
SAH	subarachnoid hemorrhage
