# Supplementary material for: Intraventricular Hemorrhage Burden and Treatment-Specific Symptomatic Vasospasm After Aneurysmal Subarachnoid Hemorrhage
Source: Diagnostics (Basel). 2026 Jul 25;16(15):2330. doi: 10.3390/diagnostics16152330 (PMC13465675; doi:10.3390/diagnostics16152330)
Supplement: Supplementary file 1 [file diagnostics-16-02330-s001.zip › diagnostics-4423641-supplementary.pdf]

**SUPPLEMENTAL MATERIAL**

# Intraventricular Hemorrhage Burden and Treatment-Specific Symptomatic Vasospasm After Aneurysmal Subarachnoid Hemorrhage

Authors: Shin-Woong Ko, Byeong Jin Ha, Sang Mook Kang, Jin Hwan Cheong, Je Il Ryu, Yu Deok Won, Myung-Hoon Han

***Corresponding Author**

Myung-Hoon Han, M.D., Ph.D.

Department of Neurosurgery, Hanyang University Guri Hospital, Guri, Gyeonggi-do, Republic of Korea

E-mail: gksmh80@gmail.com

ORCID ID: 0000-0003-1728-5017

Yu Deok Won, M.D., Ph.D.

Department of Neurosurgery, Hanyang University Guri Hospital, Guri, Gyeonggi-do, Republic of Korea

E-mail: hidma823@hanmail.net

## Original Graeb Score — Intraventricular Hemorrhage Grading System

**A** Scoring Criteria

| VENTRICLE | SCORE 0 | SCORE 1 | SCORE 2 | SCORE 3 | SCORE 4 | MAX |
|---|---|---|---|---|---|---|
| **Left lateral ventricle** *Frontal horn, body, atrium, occipital & temporal horns* | No blood | Trace | < 50% | > 50% | Expanded | 4 |
| **Right lateral ventricle** *Frontal horn, body, atrium, occipital & temporal horns* | No blood | Trace | < 50% | > 50% | Expanded | 4 |
| **Third ventricle** *Midline slit between thalami* | No blood | Blood present | Expanded | N/A | N/A | 2 |
| **Fourth ventricle** *Posterior fossa, anterior to cerebellum* | No blood | Blood present | Expanded | N/A | N/A | 2 |
| **Total Graeb Score** | Sum of all individual ventricle scores | | | | | 12 |

CSF (normal) · Trace blood · < 50% blood · > 50% blood · Filled + expanded

**B** Lateral Ventricle Scoring (0–4 per ventricle)

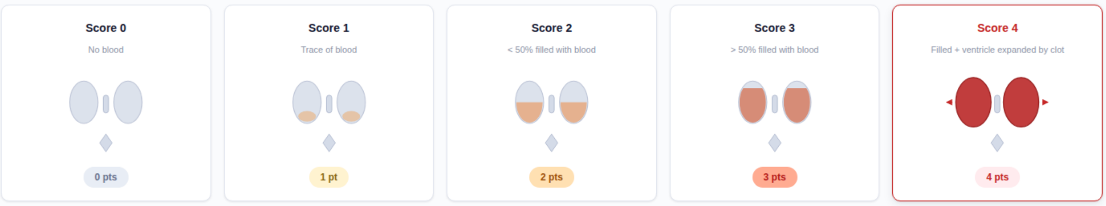


**C** Third & Fourth Ventricle Scoring (0–2 per ventricle)

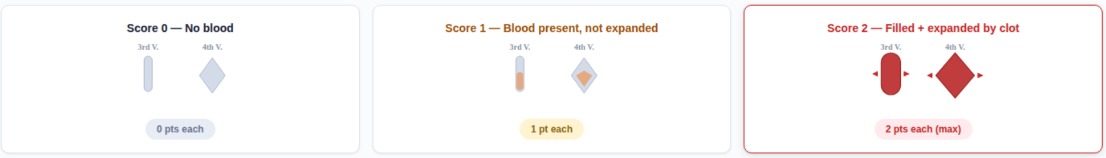


**D** Representative Composite Examples (Total Graeb Score)

*Part D presents hypothetical examples to demonstrate progressive IVH burden scoring.*

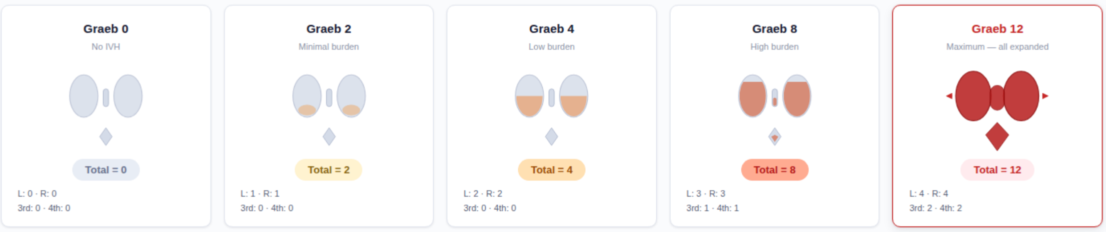


**Reference:** Graeb DA, Robertson WD, Lapointe JS, Nugent RA, Harrison PB. Computed tomographic diagnosis of intraventricular hemorrhage: etiology and prognosis. *Radiology*. 1982;143(1):91–96.

**Scoring:** Each lateral ventricle is scored 0–4 (0 = no blood, 1 = trace, 2 = less than half filled, 3 = more than half filled, 4 = filled and expanded by clot). The third and fourth ventricles are each scored 0–2 (0 = no blood, 1 = blood present, 2 = filled and expanded). Total score = sum of all four ventricles (range 0–12).

**Figure S1. Original Graeb scoring system for intraventricular hemorrhage grading.** (A) Scoring criteria for each ventricle: lateral ventricles are scored 0–4, and third and fourth ventricles are scored 0–2, yielding a total score of 0–12. (B) Schematic illustration of lateral

ventricle scoring. (C) Schematic illustration of third and fourth ventricle scoring. (D) Representative composite examples of the progressive IVH burden from Graeb 0 to 12. Abbreviations: CSF, cerebrospinal fluid; IVH, intraventricular hemorrhage.

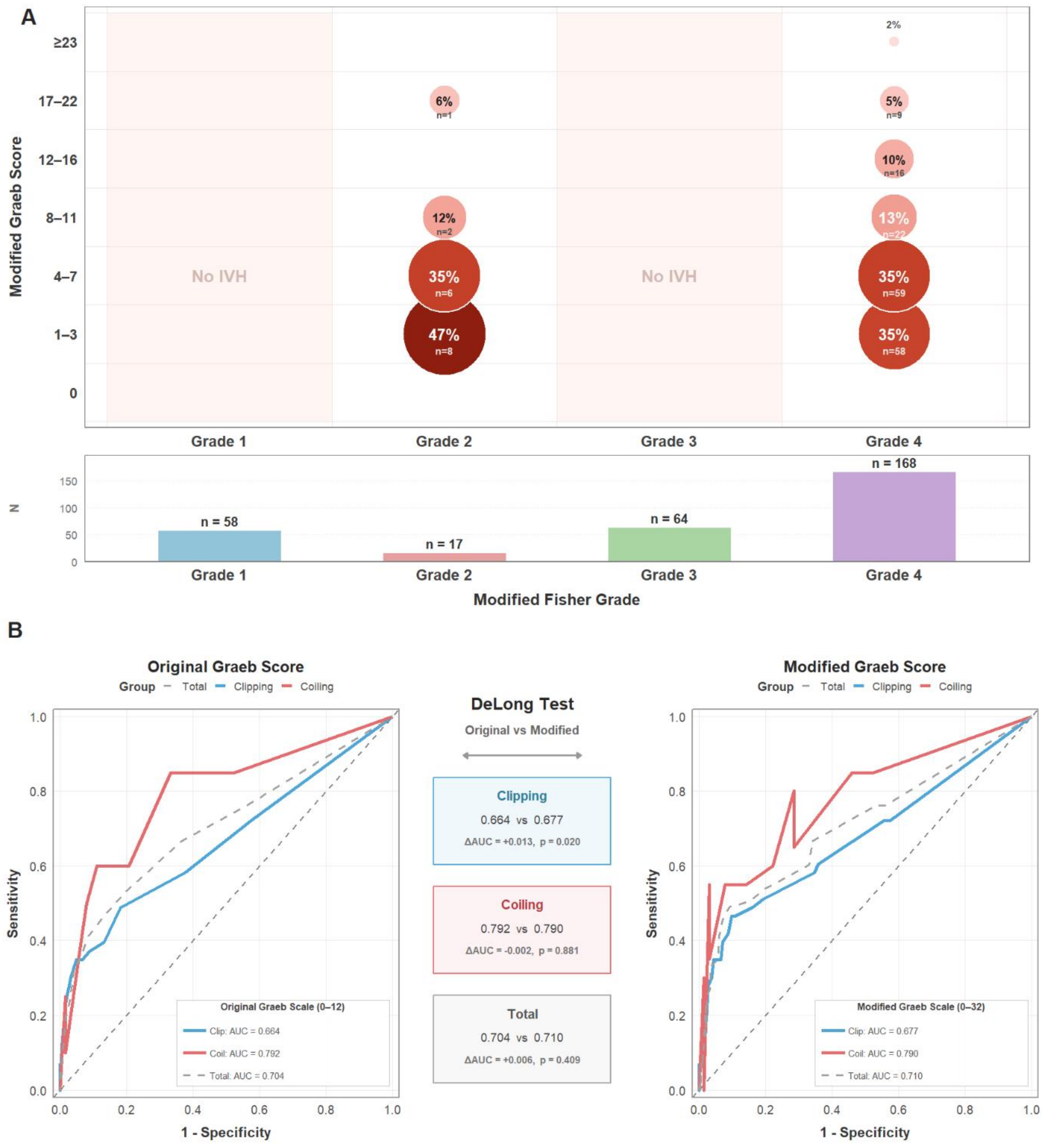


**Figure S2. Comparison of original and modified Graeb scales for the symptomatic vasospasm prediction.** (A) Distribution of modified Graeb scores (range 0–32) by modified Fisher grade. Modified Fisher grades 1 and 3 do not include IVH by definition. The bottom panel shows the total number of patients in each modified Fisher grade. (B) Receiver operating characteristic curves for the original Graeb scale (left, range 0–12) and modified

Graeb scale (right, range 0–32) in predicting symptomatic vasospasm, stratified by treatment group. The central panel displays DeLong test results comparing discriminative performance between the two scales.

Abbreviations: AUC, area under the curve; IVH, intraventricular hemorrhage.

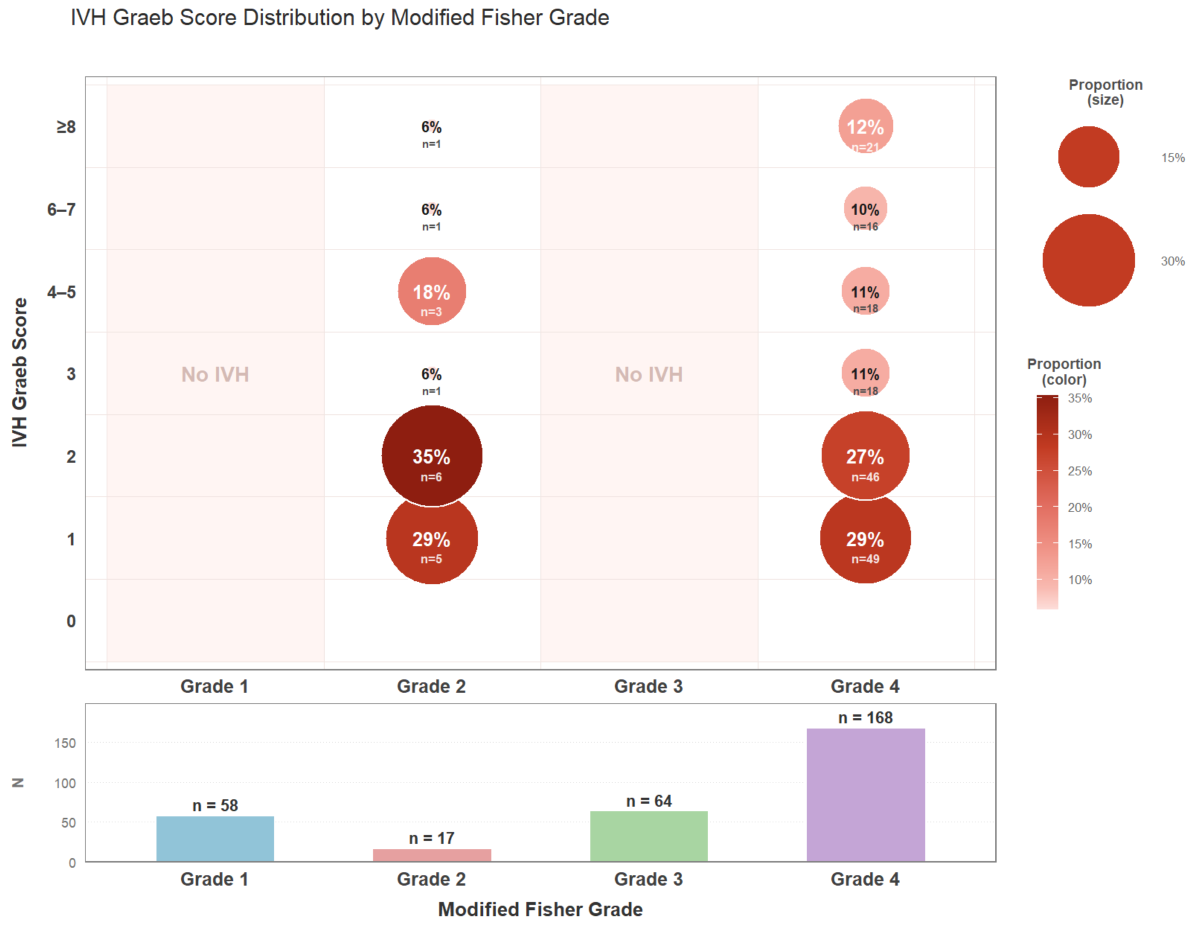


**Figure S3. Distribution of IVH Graeb scores by modified Fisher grade.** Bubble sizes and color intensity represent the proportion of patients within each modified Fisher grade. Modified Fisher grades 1 and 3 do not include IVH by definition. The bottom panel shows the total number of patients in each modified Fisher grade.

Abbreviations: IVH, intraventricular hemorrhage.

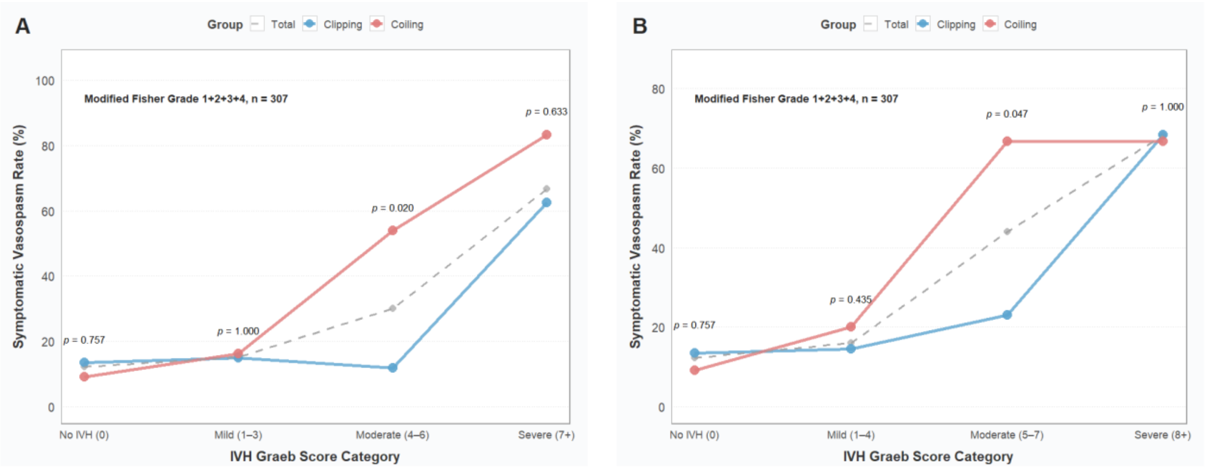


| Group | No IVH (0) | Mild (1–3) | Moderate (4–6) | Severe (7+) |
|---|---|---|---|---|
| Clipping | 12/89 (13.5%) | 14/94 (14.9%) | 2/17 (11.8%) | 15/24 (62.5%) |
| Coiling | 3/33 (9.1%) | 5/31 (16.1%) | 7/13 (53.8%) | 5/6 (83.3%) |
| Total | 15/122 (12.3%) | 19/125 (15.2%) | 9/30 (30.0%) | 20/30 (66.7%) |

| Group | No IVH (0) | Mild (1–4) | Moderate (5–7) | Severe (8+) |
|---|---|---|---|---|
| Clipping | 12/89 (13.5%) | 15/103 (14.6%) | 3/13 (23.1%) | 13/19 (68.4%) |
| Coiling | 3/33 (9.1%) | 7/35 (20.0%) | 8/12 (66.7%) | 2/3 (66.7%) |
| Total | 15/122 (12.3%) | 22/138 (15.9%) | 11/25 (44.0%) | 15/22 (68.2%) |

**Figure S4. Sensitivity analysis of symptomatic vasospasm rates using alternative IVH Graeb score categorizations.** (A) Four-tier classification: no IVH (0), mild (1–3), moderate (4–6), and severe (7+). (B) Four-tier classification: no IVH (0), mild (1–4), moderate (5–7), and severe (8+). Both analyses include all modified Fisher grades (n = 307). The *p*-values were calculated using Fisher's exact test comparing the clipping and coiling groups within each category.

Abbreviations: IVH, intraventricular hemorrhage.

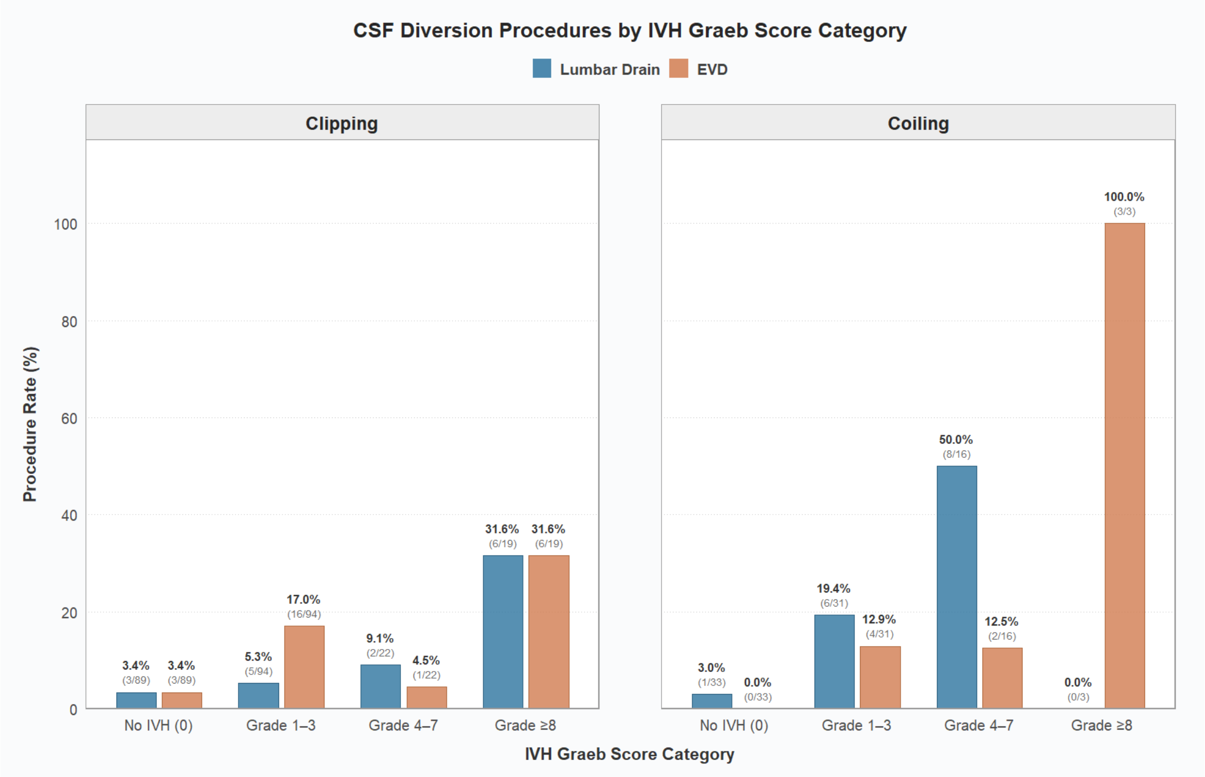


**Figure S5. Cerebrospinal fluid diversion procedures by IVH Graeb score category in the clipping and coiling groups.** Rates of lumbar drain and external ventricular drain placement are shown for each IVH Graeb score category, stratified by treatment group.

Abbreviations: CSF, cerebrospinal fluid; EVD, external ventricular drain; IVH, intraventricular hemorrhage.

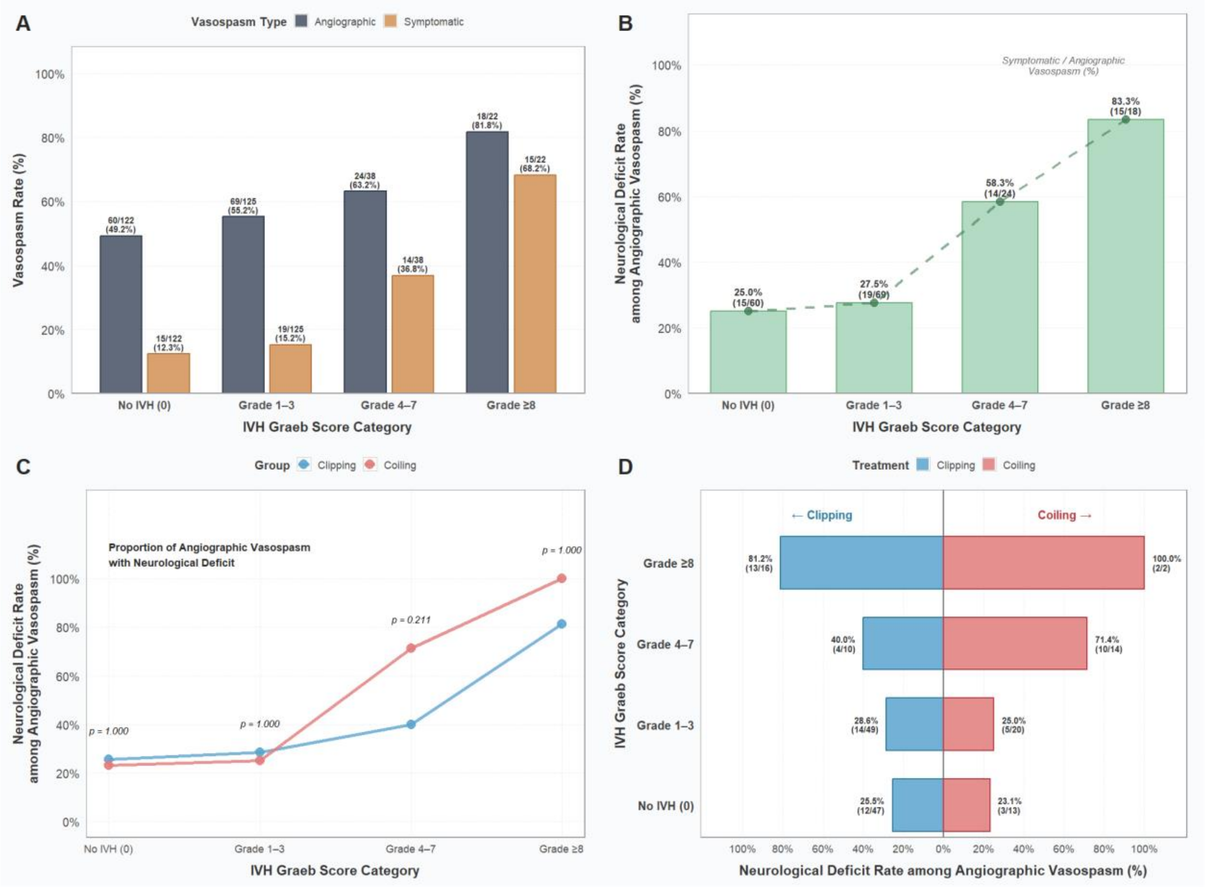


**Figure S6. Angiographic and symptomatic vasospasm rates and the symptomatic-to-angiographic vasospasm ratio by IVH Graeb score category.** (A) Rates of angiographic and symptomatic vasospasm by Graeb score category in the total cohort. (B) Symptomatic-to-angiographic vasospasm ratio by Graeb score category. (C) Comparison of the symptomatic-to-angiographic vasospasm ratio between the clipping and coiling groups by Graeb score category. (D) Butterfly plot of the same comparison as in (C). The *p*-values were calculated using Fisher's exact test (C).

Abbreviations: IVH, intraventricular hemorrhage.

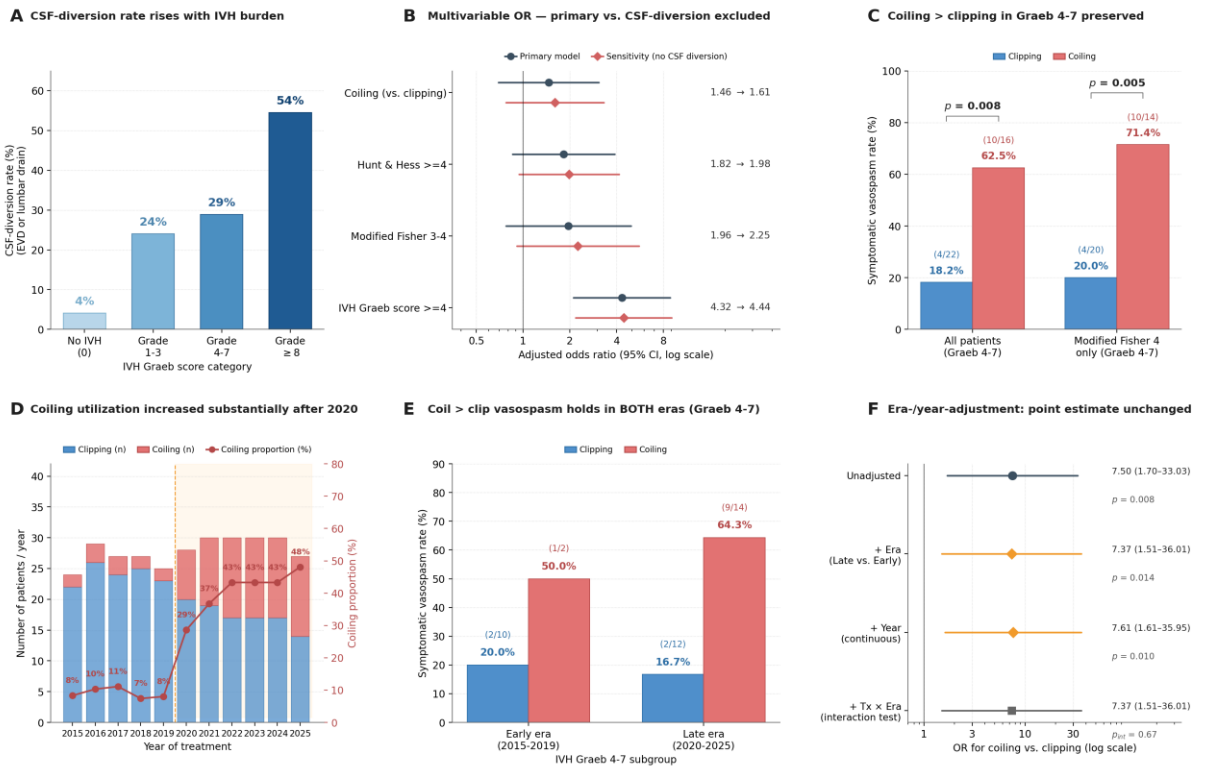


**Figure S7. Sensitivity analyses addressing CSF-diversion adjustment and the temporal shift in endovascular practice.**

(A) CSF diversion rate by IVH Graeb category, showing that the procedure is used more frequently as the IVH burden increases. (B) Multivariable adjusted ORs from the primary model versus the sensitivity model with CSF diversion were excluded. (C) Symptomatic vasospasm rate by treatment in the IVH Graeb 4–7 subgroup—the primary finding was preserved. (D) Yearly treatment volume and coiling proportion illustrating the post-2020 increase in endovascular utilization. (E) Era-stratified vasospasm rate in the Graeb 4–7 subgroup: Coiling showed a higher rate in both eras. (F) Coiling OR (vs. clipping) across four analytical strategies: unadjusted, era-adjusted, year-adjusted, and treatment × era interactions. The point estimates remain nearly identical.

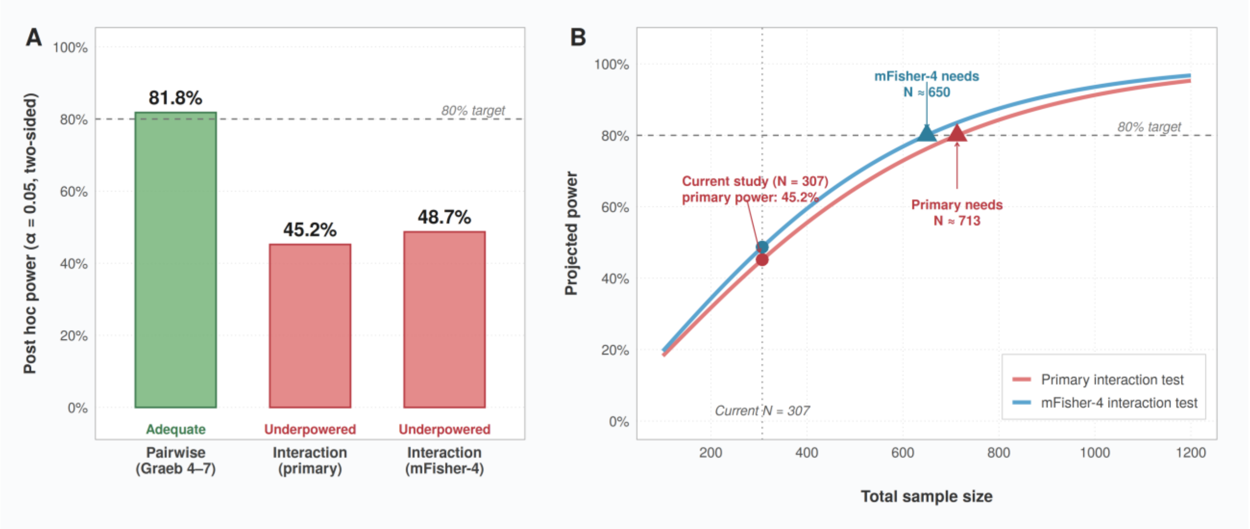


**Figure S8. Summary of the post hoc power analysis.** Panel A: observed power for each test against the 80% target. Panel B: sample-size trajectory needed to reach 80% power on the interaction, with the current N = 307 highlighted.

**Table S1. Multivariable logistic regression for symptomatic vasospasm — primary model vs. sensitivity model excluding CSF diversion (n = 307).**

| **Predictor** | **Primary model** (with CSF diversion) | **Sensitivity model** (CSF diversion excluded) |
|---|---|---|
| **IVH Graeb score ≥ 4** | **4.32 (2.10–8.87), p<0.001** | **4.44 (2.19–9.13), p<0.001** |
| Diabetes mellitus | 2.28 (0.79–6.55), p=0.127 | 2.56 (0.90–7.11), p=0.073 |
| Modified Fisher grade 3–4 | 1.96 (0.78–4.95), p=0.154 | 2.25 (0.96–6.00), p=0.079 |
| Hunt & Hess grade ≥ 4 | 1.82 (0.86–3.89), p=0.119 | 1.98 (0.93–4.14), p=0.072 |
| CSF diversion (EVD/lumbar) | 1.76 (0.78–3.96), p=0.172 | — (removed) |
| Hypertension | 1.53 (0.76–3.08), p=0.232 | 1.57 (0.78–3.15), p=0.201 |
| **Coiling (vs. clipping)** | **1.46 (0.70–3.06), p=0.317** | **1.61 (0.77–3.31), p=0.200** |
| Smoking | 1.37 (0.41–4.64), p=0.612 | 1.60 (0.48–5.22), p=0.437 |
| Intracerebral hemorrhage | 1.35 (0.66–2.75), p=0.410 | 1.43 (0.70–2.87), p=0.323 |
| Age (per year) | 1.01 (0.98–1.04), p=0.420 | 1.01 (0.99–1.04), p=0.339 |
| Alcohol use | 0.85 (0.30–2.46), p=0.770 | 0.82 (0.27–2.25), p=0.711 |
| Female sex | 0.63 (0.30–1.32), p=0.220 | 0.66 (0.32–1.38), p=0.272 |
| Posterior location | 0.45 (0.11–1.86), p=0.269 | 0.63 (0.15–2.15), p=0.486 |

Abbreviations: CI, confidence interval; CSF, cerebrospinal fluid; EVD, external ventricular drain; IVH, intraventricular hemorrhage; OR, odds ratio.

**Table S2. Baseline characteristics of coiling patients by era.**

| Variable | Early era Coiling (n = 12) | Late era Coiling (n = 71) | All coiling (n = 83) | *p* |
|---|---|---|---|---|
| Age (years, mean ± SD) | 54.0 ± 14.7 | 56.7 ± 13.0 | 56.3 ± 13.2 | 0.411 |
| Female sex | 6 (50.0%) | 39 (54.9%) | 45 (54.2%) | 0.765 |
| Posterior aneurysm location | 2 (16.7%) | 8 (11.3%) | 10 (12.0%) | 0.633 |
| Hunt & Hess grade ≥ 4 | 2 (16.7%) | 13 (18.3%) | 15 (18.1%) | 1.000 |
| Modified Fisher grade 3–4 | 7 (58.3%) | 54 (76.1%) | 61 (73.5%) | 0.287 |
| IVH Graeb score, median [IQR] | 2 [0–2] | 1 [0–3] | 1 [0–3] | 0.702 |
| IVH Graeb score ≥ 4 | 2 (16.7%) | 17 (23.9%) | 19 (22.9%) | 0.724 |
| Intracerebral hemorrhage | 2 (16.7%) | 9 (12.7%) | 11 (13.3%) | 0.657 |
| Hydrocephalus on admission | 8 (66.7%) | 41 (57.7%) | 49 (59.0%) | 0.753 |
| Hypertension | 4 (33.3%) | 19 (26.8%) | 23 (27.7%) | 0.730 |
| Diabetes mellitus | 1 (8.3%) | 6 (8.5%) | 7 (8.4%) | 1.000 |
| Smoking | 0 (0.0%) | 3 (4.2%) | 3 (3.6%) | 1.000 |

| **Variable** | **Early era** Coiling (n = 12) | **Late era** Coiling (n = 71) | **All coiling** (n = 83) | ***p*** |
|---|---|---|---|---|
| Alcohol use | 1 (8.3%) | 2 (2.8%) | 3 (3.6%) | 0.378 |
| Symptomatic vasospasm | 2 (16.7%) | 18 (25.4%) | 20 (24.1%) | 0.721 |

Mann–Whitney U for continuous variables and Fisher's exact test for categorical variables.

Abbreviations: IQR, interquartile range; IVH, intraventricular hemorrhage; SD, standard deviation.

**Table S3. Sensitivity analyses for the temporal shift in treatment practice (n = 307)**

| **Analysis** | **Comparison / group** | **OR (95% CI)** | ***p* value** |
|---|---|---|---|
| ***Panel A. Utilization and era-stratified event rates (IVH Graeb 4–7)*** | | | |
| Coiling utilization by era | Early: 12/132 (9.1%) vs. Late: 71/175 (40.6%) | — | **<0.001** |
| Vasospasm rate — Early era | Clip 2/10 (20.0%) vs. Coil 1/2 (50.0%) | 4.00 (0.20–79.4) | 0.454 |
| Vasospasm rate — Late era | Clip 2/12 (16.7%) vs. Coil 9/14 (64.3%) | **9.00 (1.42–57.1)** | **0.021** |
| ***Panel B. Coiling effect across adjustment strategies (IVH Graeb 4–7, n = 38)*** | | | |
| Unadjusted | Coiling vs. clipping | **7.50 (1.70–33.0)** | **0.008** |
| + Era (Late vs. Early) | Coiling vs. clipping, adjusted | **7.37 (1.51–36.0)** | **0.014** |
| + Year (continuous) | Coiling vs. clipping, adjusted | **7.61 (1.61–35.9)** | **0.010** |
| Tx × Era interaction (LRT) | Test for heterogeneity between eras | — | 0.667 ($p_{int}$) |

Fisher's exact test for 2 × 2 event-rate comparisons; logistic regression for adjusted analyses.

Abbreviations: CI, confidence interval; LRT, likelihood-ratio test; OR, odds ratio.

**Table S4. Post hoc power analysis for the Graeb 4–7 pairwise comparison and the treatment × Graeb-category interaction (n = 307)**

| Analysis | Parameters / observed effect | Result | *p* value |
|---|---|---|---|
| ***Panel A. Pairwise subgroup comparison — Graeb 4–7*** | | | |
| **Two-proportion z-test** | **$p_1$ = 18.2% (4/22); $p_2$ = 62.5% (10/16); Cohen's h = 0.94** | **Power = 81.8%** | — |
| **Fisher's exact test (Monte Carlo, 30,000 replicates)** | **Same as above** | **Power = 78.7%** | — |
| Minimum detectable effect (MDE) at 80% power | Coiling rate detectable at ≥ 61.4% (OR ≥ 7.17) | — | — |
| ***Panel B. Treatment* × *Graeb-category interaction*** | | | |
| **Interaction test — primary model (n = 307)** | **LRT $\chi^2$ = 7.20, df = 3; \|Z\|_obs = 1.84** | **Power = 45.2%** | **0.066** |
| **Interaction test — modified Fisher 4 subgroup (n = 168)** | **LRT $\chi^2$ = 5.84, df = 2; \|Z\|_obs = 1.93** | **Power = 48.7%** | **0.054** |
| ***Panel C. Sample size required for 80%*** | | | |

| Analysis | Parameters / observed effect | Result | *p* value |
|---|---|---|---|
| ***power on the interaction*** | | | |
| Primary model — scaling factor | $(z_{\alpha/2} + z_{\beta})^2 / \lvert Z_{obs}\rvert^2$ = 2.32× | ≈ 713 total | — |
| Modified Fisher 4 subgroup — scaling factor | $(z_{\alpha/2} + z_{\beta})^2 / \lvert Z_{obs}\rvert^2$ = 2.11× | ≈ 650 total | — |

Post-hoc power was computed using Cohen's h and the two-proportion z-approximation (R package *pwr*) and confirmed by Fisher's exact test using 30,000 Monte Carlo replicates. Interaction test power was computed at the observed effect size. Abbreviations: LRT, likelihood-ratio test; MDE, minimum detectable effect; OR, odds ratio.
